# Systematic review of Chinese medicine for the treatment of atherosclerosis

**DOI:** 10.3389/fcvm.2025.1671008

**Published:** 2025-11-03

**Authors:** Cuiyao Tang, Bo Liu, Ying Zhang, Mengyang Long, Wei Zheng, Jing Lu, Han Li, Zihui Xu, Yunqiao Wang

**Affiliations:** Department of Integrative Medicine, The Second Affiliated Hospital of Army Medical University (Third Military Medical University), Chongqing, China

**Keywords:** Chinese medicine, atherosclerosis, inflammation, lipid metabolism, glucose metabolism

## Abstract

**Introduction:**

Atherosclerosis (AS) is a chronic inflammatory metabolic disease strongly associated with risk factors, including hypertension, hyperlipidemia, hyperglycemia, and hyperuricemia. AS serves as the pathological foundation for numerous cardiovascular diseases (CVDs), and it remains a major threat to global health. However, the underlying mechanisms driving AS development are incompletely understood. Elucidating the pathogenesis and key influencing factors of AS is critical for identifying novel preventive strategies and therapeutic approaches.

**Methods:**

We searched PubMed and Web of Science for relevant studies. We selected relevant English research articles published between 2012 and 2024. Afterward,we analyzed and summarized the pharmacological effects and molecular mechanisms of these Chinese medicines.

**Results:**

Through our search and exclusion criteria, a total of 116 preclinical studies and 6 clinical research articles were found.

**Discussion:**

Traditional Chinese medicine (TCM), with over 2000 years of clinical application, offers a rich source of potential interventions. Integrating modern medical technologies allows for the reevaluation of TCM from a natural compound perspective. This review comprehensively summarizes the mechanisms by which single herbal medicines (SHMs) and their derived natural compounds (NCs) exert effects against AS on the basis of preclinical evidence and analysis of seven selected double-blind, randomized, placebo-controlled clinical trials (RCTs).

## 1 Introduction

Atherosclerosis (AS) is a primary pathological driver of cardiovascular diseases (CVDs) worldwide. The pathological features of atherosclerosis include endothelial cell dysfunction, lipoprotein deposition, inflammatory cell differentiation, vascular smooth muscle cell proliferation and death, etc. (1, 2). Moreover, CVDs are a significant cause of morbidity and mortality, especially in China, on the basis of the outcomes of *ANNUAL REPORT ON CARDIOVASCULAR HEALTH AND DISEASES IN China (2023)* (3).

For AS, artificially synthesized drugs, such as statins, nicotine, angiotensin receptor blockers, antioxidants, antiplatelets and anticoagulants, are commonly used in therapy. However, their toxic side effects limit their clinical use to some extent. While statins and antiplatelet therapies can lower lipid levels and inhibit thrombosis, they struggle to comprehensively target multiple pathological pathways, such as those involved in endothelial repair and inflammation regulation. Aspirin (4), although widely used in antiplatelet therapy to suppress thrombosis, has limited efficacy in preventing plaque progression and vascular events in asymptomatic carotid atherosclerosis patients. The long-term use of these drugs also has significant side effects, including hepatotoxicity and gastrointestinal bleeding.

In this context, Chinese medicine for the treatment of AS is gradually gaining widespread attention because of its advantages of being safe at specific doses, effective, less toxic, and inexpensive, and exploring efficient and safe Chinese medicine has become a new direction for the treatment of AS.

The Chinese medicines mentioned here include single herbal medicines (SHMs) and the natural compounds (NCs) derived from them. In addition to natural compounds, herbs have also been used for more than 2000 years. Modern medicine has also helped us understand TCM from the perspective of natural compounds. In this review, we searched PubMed and Web of Science for relevant studies. We selected relevant English research articles published between 2012 and 2024. Afterward, we analyzed and summarized the pharmacological effects and molecular mechanisms of these Chinese medicines.

## 2 Methods

### 2.1 Literature searches

**Figure 1:**
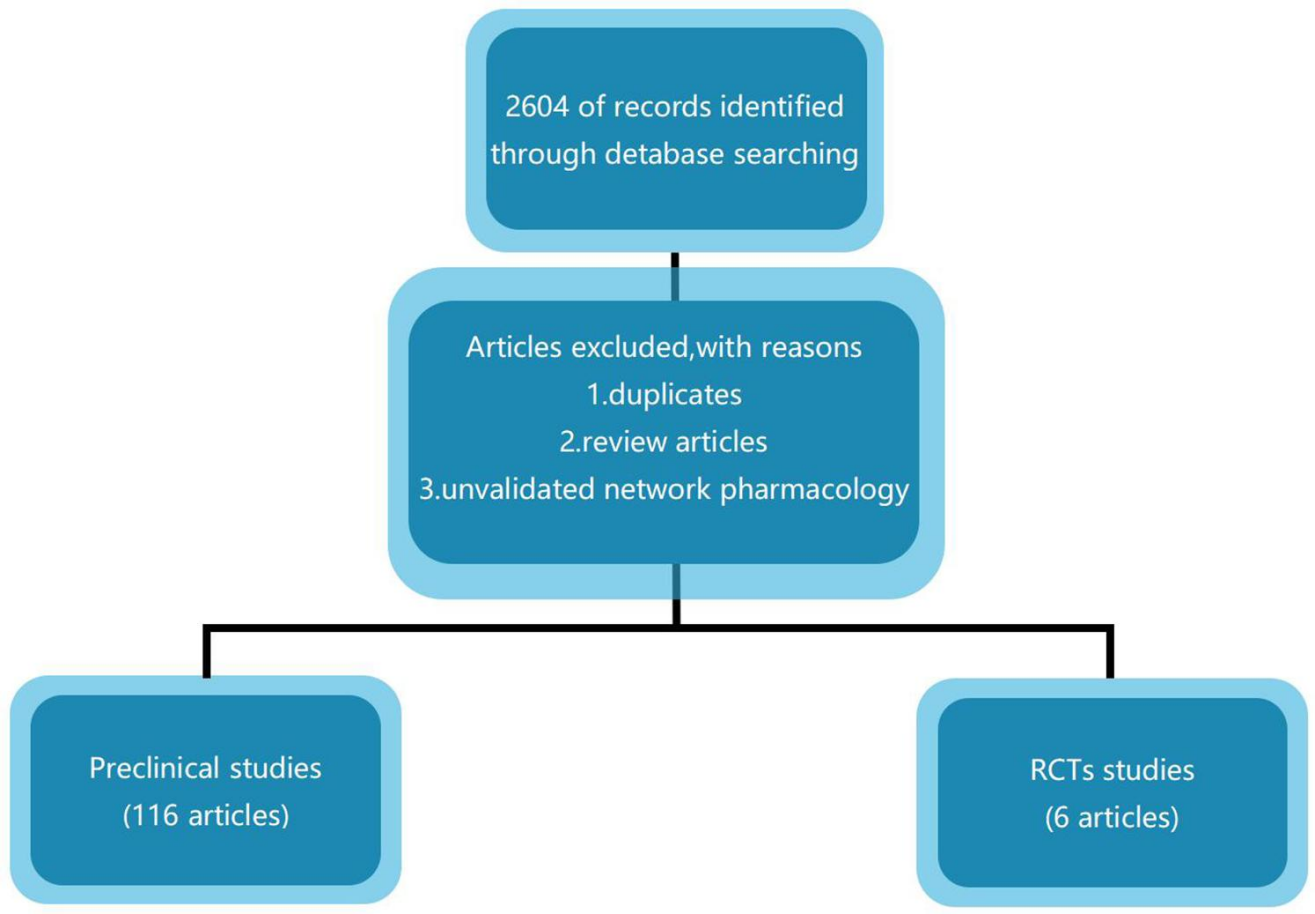
Study flow diagram.

We searched PubMed and Web of Science for relevant English research articles published between 2012 and 2024. There were no restrictions on the study type during the search process. The databases were searched via the following terms: [“traditional Chinese medicine (TCM)” OR “Chinese medicine” OR “herbal medicine” AND “atherosclerosis”]. To ensure the systematic integrity of the literature retrieval, each database's interface was used to retrieve subject words combined with keywords and free words. We selected all preclinical studies, both *in vivo* and *in vitro*, on the antiatherosclerotic mechanism of NC and HM and identified all proven targets. We also evaluated and summarized the retrieved RCTs (Figure 1).

### 2.2 Criteria for inclusion and exclusion

According to discussions between researchers and the advice of experts, the following inclusion and exclusion criteria were developed to identify relevant studies.

(1) Types of participants or models: Atherosclerosis. (2) Types of interventions: single herbal medicines or the natural compounds. (3) Types of comparisons: no restrictions. (4) Types of outcomes: no restrictions. (5) Types of research design: animal experiments, cell experiments, RCT, cohort studies, case-control studies, case series, case reports.

#### 2.2.1 Exclusion criteria

(1) Duplicates. (2) Review articles. (3) Unvalidated network pharmacology.

We searched PubMed and Web of Science for relevant English research articles published between 2012 and 2024. The databases were searched via the following terms: [“traditional Chinese medicine (TCM)” OR “Chinese medicine” OR “herbal medicine” AND “atherosclerosis”]. To ensure the systematic integrity of the literature retrieval, each database's interface was used to retrieve subject words combined with keywords and free words. We selected all preclinical studies, both *in vivo* and *in vitro*, on the antiatherosclerotic mechanism of NC and HM and identified all proven targets. We also evaluated and summarized the retrieved RCTs (Figure 1).

## 3 Results

### 3.1 Mechanisms of Chinese medicine for treating AS

**Figure 2:**
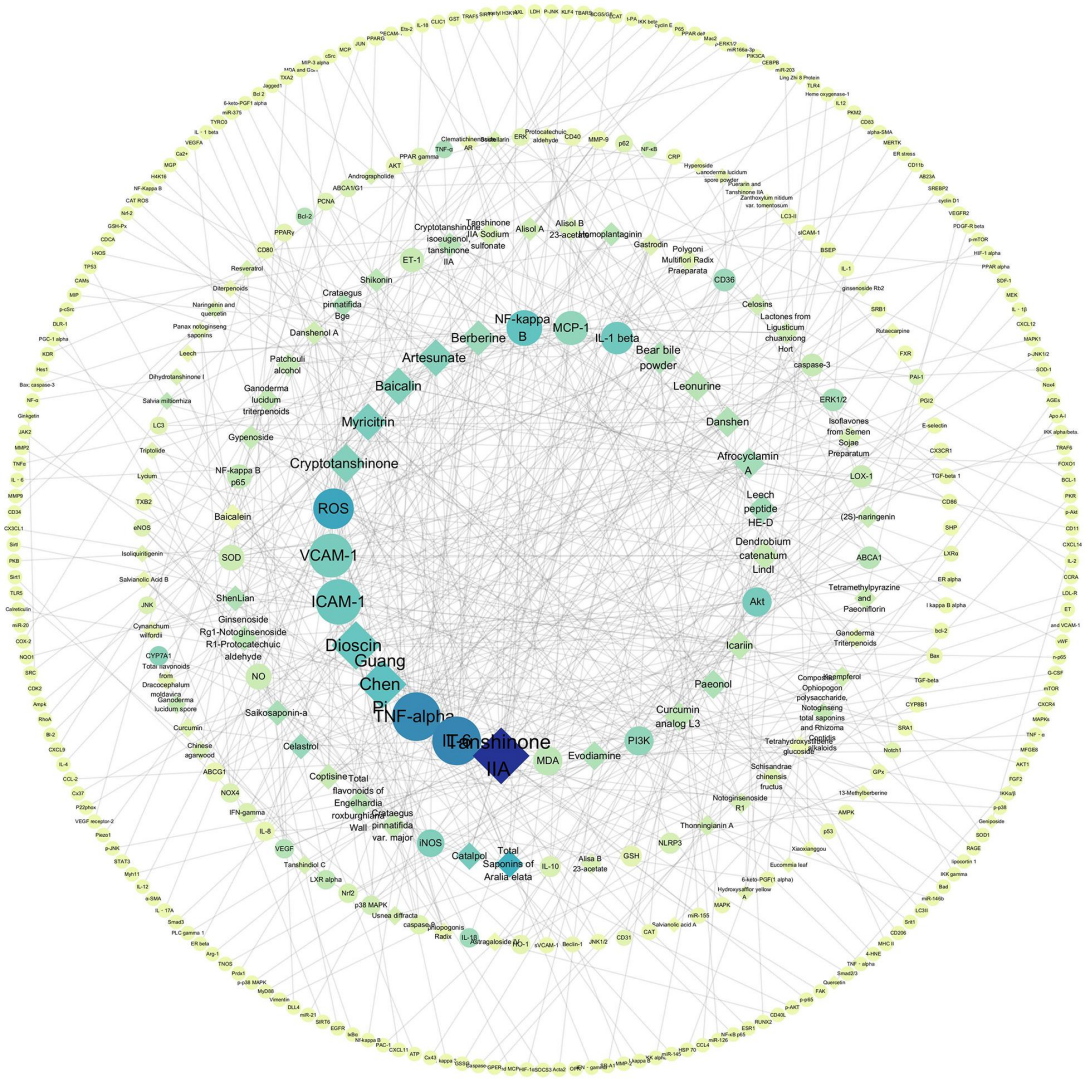
Target-Chinese medicine component association network.

Through our search and exclusion criteria, a total of 116 preclinical studies were found, and the validated targets were summarized (Table 1, Figure 2).

**Table 1:** The mechanisms of Chinese medicine for treating AS.

Name of Chinese Medicine	Type of Chinese Medicine		Applied model	Type of model		Reported targets
	NC	SHM		Animal	Cell	
Cryptotanshinone (5)	+		HUVECs		+	TNF-alpha, sICAM-1, sVCAM-1, MCP-1
Paeonol (6)	+		HUVECs		+	LOX-1, Bcl-2, caspase-3, p38 MAPK
ginsenoside Rb2 (7)	+		HUVECs		+	Smad3, NF-kappa B
Clematichinenoside AR (8)	+		RAW264.7		+	ABCA1, ABCG1, NLRP3
Notoginsenoside R1 (9)	+		HUVECs		+	MGP, MCP-1, ICAM-1, JNK, NF-kappa B
Evodiamine (10)	+		VSMCs		+	PPAR gamma
Afrocyclamin A (11)	+		VSMCs		+	PCNA, p38 MAPK, TNF-alpha, IL-1 beta, IL-6, VCAM-1, MDA, ET-1, SOD, GSH, NO
Saikosaponin-a (12)	+		THP-1		+	DLR-1, CD36, ATP, PPAR gamma, PI3K, AKT, NF-kappa B, NLRP3
Icariin (13)	+		HUVECs		+	ICAM-1, VCAM-1, E-selectin,
Gypenoside	+		THP-1		+	LC3-II, p62, Srit1, FOXO1
Paeonol (14)	+		HUVECs		+	p53, acetyl H3K14, H4K16, Sirtl
Salvianolic Acid B (15)	+		RAW 264.7		+	HO-1, NO, iNOS
(2S)-naringenin (16)	+		VSMCs		+	PDGF-R beta, ERK1/2, PI3 K, Akt, PKB, PLC gamma 1
Paeonol (17)	+		HUVECs		+	Beclin-1, LC3II
Curcumin (18)	+		THP-1		+	ABCA1, AMPK, SIRT1, LXR alpha
Rutaecarpine (19)	+		THP-1		+	Cx37, Cx43
			HUVECs		+	
Protocatechuic aldehyde (20)	+		VSMCs		+	PI3K, Akt, MAPK
13-Methylberberine (21)	+		HUVECs		+	ROS, NLRP3
Celastrol (22)	+		VSMCs		+	ABCA1, LXR alpha
Triptolide (23)	+		HUVECs		+	NF-kappa B p65
Gypenoside (24)	+		ApoE−/− mice	+		PI3K, Akt, Bad
Hydroxysafflor yellow A (25)	+		VSMCs		+	Akt, Heme oxygenase-1
Resveratrol (26)	+		HUVECs		+	VEGF, KDR, VEGF receptor-2
Leech peptide HE-D (27)	+		RAW264.7		+	IKK alpha, IKK gamma, TRAF6, TLR4, TRAF5, NF-Kappa B, iNOS, TNF-alpha, Arg-1, IL-10
Total flavonoids from Dracocephalum moldavica (28)	+		VSMCs		+	PCNA, NF-kappa B p65, ICAM-1, VCAM-1
Protocatechuic Aldehyde (29)	+		HUVECs		+	Caspase-3
Tanshindiol C (30)	+		RAW264.7		+	Nrf2, Sirt1, Prdx1, ABCA1
Tetramethylpyrazine and Paeoniflorin (31)	+		HUVECs		+	CD31, VEGF, VEGFR2, Notch1, Jagged1, Hes1
Lycium (32)	+		VSMCs		+	PI3K, Akt, miR-145
Shikonin (33)	+		VSMCs		+	NF-kappa B, PI3K, cyclin D1, cyclin E, Bcl 2, Bax; caspase-3, caspase-9
Hyperoside (34)	+		VSMCs		+	LOX-1, ERK
Danshenol A (35)	+		HUVECs		+	ROS, NOX4, IKK beta, I kappa B alpha, NF-kappa B p65, TNF-alpha, ICAM-1
Total Saponins of Aralia elata (36)	+		HUVECs		+	TNF-alpha, NF-kappa B, IL-6, MCP-1, and VCAM-1, PI3K, Akt, Bcl-2, Bax
			THP-1		+	
Icariin (37)	+		Wistar rats	+		IL-6, TNF-alpha, SOD, MDA, p-p38 MAPK
Tanshinone IIA (38)	+		SD Rats	+		CYP7A1, LDL-R, SREBP2, LCAT, ABCA1, CD36
			THP-1		+	
Tetrahydroxystilbene glucoside (39)	+		SD Rats	+		Calreticulin, Vimentin, HSP 70, lipocortin 1, Apo A-I
Baicalin (40)	+		SD Rats	+		TBARS, SOD, GSH-Px
Composition of Ophiopogon polysaccharide, Notoginseng total saponins and Rhizoma Coptidis alkaloids (41)	+		New Zealand white rabbits	+		p-JNK, Caspase-3, Bcl-2, RAGE, AGEs
Ling Zhi 8 Protein (42)	+		New Zealand white rabbits	+		IL-1 beta
Ganoderma lucidum triterpenoids	+		Japanese big-ear white rabbits	+		LOX-1, ROS, MDA, NF-kappa B p65, Notch1, DLL4, TNF-alpha
Polysaccharides (43)	+		HUVECs		+	
			THP-1		+	
Scutellarin (44)	+		New Zealand white rabbits	+		SOD1, Nox4, ROS
			SD Rats	+		
			HUVECs		+	
Leonurine (45)	+		New Zealand white rabbits	+		PECAM-1, sVCAM-1, sICAM-1, IL-6, TNF-alpha, MCP-1, iNOS, MMP-9, CAT, SOD-1, GPx
Catalpol (46)	+		ApoE−/− mice	+		ER alpha, ROS, iNOS, eNOS, CRP, IL-1 beta, TNF-α, IL-10, CD11b
			J774A-1		+	
Tanshinone IIA (47)	+		ApoE−/− mice	+		KLF4, miR-375
Coptisine (48)	+		ApoE−/− mice	+		IL-6, IL-1 beta, TNF-alpha, NF-kappa B p65, VCAM-1, ICAM-1, p-p38, p-JNK1/2
Berberine	+		ApoE−/− mice	+		IL-1 beta, TNF-alpha, NF-kappa B p65, i-NOS, ICAM-1, IL-6
8-cetylberberine (49)	+					
Kaempferol (50)	+		ApoE−/− mice	+		GPER, PI3K, AKT, Nrf2, ROS
Alisa B 23-acetate (51)	+		Ldlr−/− mice	+		CYP7A1, CYP8B1, SHP, FXR, BSEP
Celastrol (52)	+		ApoE−/− mice	+		LOX-1, ROS, iNOS, NO, TNF-alpha, IL-6
			RAW264.7		+	
Isoflavones from Semen Sojae Preparatum (53)	+		ApoE−/− mice	+		ET-1, LDH, SOD, MDA, 2, HO-1, NQO1,
Ganoderma Triterpenoids (54)	+		BALB/cC mice	+		VCAM-1, TNF-alpha, IL-6, MCP-1, ET-1
			HUVECs		+	
Artesunate (55)	+		ApoE−/− mice	+		TNF-alpha, IL-6, IL-8, MCP-1
			HUVECs		+	
Baicalin	+		HUVECs		+	CAMs, ROS, NF-kappa B
Baicalein	+					
Wogonin (56)	+					
Gastrodin (57)	+		C57BL/6J mice	+		TNF-alpha, IL-1 beta, IL-6, IL-8, IL-10, VCAM-1,
			VSMCs		+	
ShenLian (58)	+		THP-1		+	alpha-SMA, TGF-beta, TGF-beta, iNOS, JAK2, STAT3, SOCS3, Smad2/3
			RAW264.7		+	
			ApoE−/− mice	+		
Baicalin	+		ApoE−/− mice	+		CD11, CD83, CD80, CD86, TNFα, IL-12
Geniposide (59)	+					
Baicalin (60)	+		ApoE−/− mice	+		VCAM-1, MCP-1, IL-6
Cryptotanshinone (61)	+		ApoE−/− mice	+		LOX-1, MMP-9, ROS, NOX4, VCAM-1, ICAM-1, NF-kappa B, IL-1 beta, IL-6, IL-17A, IFN-gamma, TNF-alpha
			HUVECs		+	
Berberine (62)	+		ApoE−/− mice	+		TNF-alpha, IL-6, IL-1 beta, IFN-gamma, MCP, MIP, Ampk, Nf-kappa B
Homoplantaginin	+		ApoE−/− mice	+		ICAM-1, VCAM-1, ROS, ERK, NF-kappa B, Nrf2, HO-1
Dihydrohomoplantagin (63)	+		HUVECs		+	
Panax notoginseng saponins (64)	+		ApoE−/− mice	+		VEGF, NOX4, CD34
Astragaloside IV (65)	+		ApoE−/− mice	+		PAC-1, CD40l, CXCR4, SDF-1,
Myricitrin (66)	+		ApoE−/− mice	+		ROS, NO, p53, caspase-3, MAPK, Bl-2, Bax, eNOS, MDA, 4-HNE, SOD, CAT, ERK1/2, ERK1/2, JNK
			HUVECs		+	
Thonningianin A (67)	+		ApoE−/− mice	+		Ca2+, AMPK, IL-1, ROS, NLRP3
			HUVECs		+	
Alisol B 23-acetate (68)	+		ApoE−/− mice	+		MHC II, CD80, CD86, IL12, IFN-gamma, IL-10, TGF-beta 1
			BMDCs		+	
Celosins (69)	+		ApoE−/− mice			LC3, CD36, SR-A1, ABCA1, ABCG1, BCL-1
Patchouli alcohol (70)	+		ApoE−/− mice			Mac2, MCP-1, iNOS, IL-1 beta, IL-6, CXCL9, CXCL11
Alisol A (71)	+		ApoE−/− mice	+		ICAM-1, IL-6, MMP-9, PPAR alpha, PPAR delta, NF-kappa B, I kappa B alpha
			HepG2		+	
Artesunate (72)	+		ApoE−/− mice	+		iNOS, TNF-alpha, IL-4, IL-6, IL-1 beta, CD80, CD206, TGF-beta 1, HIF-1 alpha, P65, NF-kappa B
			RAW 264.7		+	
Icariin (73)	+		ApoE−/− mice	+		CX3CR1, CX3CL1
			RAW 264.7		+	
Total flavonoids of Engelhardia roxburghiana Wall (74)	+		ApoE−/− mice	+		IL-1 beta, AKT, p-AKT, mTOR, p-mTOR, Beclin-1, LC3-II, p62
			THP-1		+	
Lactones from Ligusticum chuanxiong Hort (75)	+		ApoE−/− mice	+		ICAM-1, VCAM-1, TNF-alpha, NF-kappa B, CD31, MCP-1
			HUVECs		+	
Tanshinone IIA (76)	+		ApoE−/− mice	+		CD40, G-CSF, IFN-gamma, IL-1 beta, IL-6, MCP-1, MIP-3 alpha, TNF-alpha, VEGF, CCL-2, CD40, MMP-2, miR-146b, miR-155
Dioscin (77)	+		Ldlr−/− mice	+		MDA, GSH, MDA and GSH, ROS, NOX4, P22phox, I kappa 3, p-p65, n-p65, ICAM-1, VCAM-1, caspase-3, caspase-9, bcl-2, PGC-1 alpha, ER alpha, ER beta, I kappa B, caspase-3, caspase-9, bcl-2, LC3
			HAECs		+	
Ginsenoside Rg1-Notoginsenoside R1-Protocatechuic aldehyde (78)	+		ApoE−/− mice	+		ET-1, eNOS, TXA2, PGI2, Piezo1, PI3 K, Akt, FAK
			HUVECs		+	
Curcumin analog L3 (79)	+		ICR mice	+		GSH, SOD, GPx, MDA, GST, CAT ROS, LOX-1, NO, TNOS, iNOS
Tanshinone IIA Sodium sulfonate (80)	+		ApoE−/− mice	+		ROS, MDA, TNF-alpha, IL-6, ICAM-1, VCAM-1, CLIC1
			HUVECs		+	
Dendrobium catenatum Lindl (81)		+	Zebrafish	+		ROS, SOD, MDA, GSH, GSSG, NO, ET-1, PGI2, ICAM-1, VCAM-1
			EA.hy926		+	
Guang Chen Pi (82)		+	RAW264.7		+	SRA1, CD36, PPAR gamma, LXR alpha, SRB1, ABCG1, p38 MAPK, ERK1/2, JNK1/2, NF-kappa B p65, IKK alpha/beta.
Danshen (83)		+	HUVECs		+	VCAM-1, ICAM-1, CD40, IL-6, IL-8, MCP-1
Ophiopogonis Radix (84)		+	VSMCs		+	ROS, NO, ICAM-1, VCAM-1
Crataegus pinnatifida Bge (85)		+	SD Rats	+		vWF, VCAM-1, ICAM-1, t-PA, 6-keto-PGF1 alpha, PAI-1, TXB2
			HUVECs		+	
Crataegus pinnatifida var. major (86)		+	SD Rats	+		IL-1 beta, IL-8, NO, ET, 6-keto-PGF (1 alpha), TXB2, IL-18, CRP
Usnea diffracta (87)		+	SD Rats	+		TLR5, MyD88, NF-kappa B, IL-10
Ganoderma lucidum spore (88)		+	Japanese rabbits	+		LXR alpha, CYP7A1, ABCA1/G1, ABCG5/G8
Schisandrae chinensis fructus (89)		+	SD Rats	+		Nrf-2, HO-1, ET-1,6-keto-PGF (1 alpha), TXB2
Cynanchum wilfordii (90)		+	ApoE−/− mice	+		ET-1, VCAM-1, ICAM-1, E-selectin
Eucommia leaf (91)		+	ApoE−/− mice	+		IL-1, TNF-alpha
Danshen (92)		+	ApoE−/− mice	+		LC3, p62, caspase-3, caspase-9, IL-6
			RAW 264.7		+	
			HUVECs		+	
Polygoni Multiflori Radix Praeparata		+	ApoE−/− mice	+		IL-6, TNF-alpha, VCAM-1, MCP-1, ICAM-1, CCRA
2,3,5,4′-Tetrahydroxy-stilbene-2-O-beta-D-glucoside (TSG) (93)	+					
Xiaoxianggou (94)		+	ApoE−/− mice	+		miR-203, Ets-2
Leech (95)		+	ApoE−/− mice	+		ICAM-1, MCP-1, NF-kappa B
			EA.hy926		+	
Zanthoxylum nitidum var. tomentosum Toddalolactone (96)		+	ICR mice	+		PAI-1, CCL4,
Total red ginseng saponin extracts (TGS) (97)	+					
Bear bile powder (98)		+	ApoE−/− mice	+		IL-2, IL-6, TNF-alpha, IFN-gamma, MDA, GSH, SOD, ROS, miR-20, miR-21, miR-126, miR-155
Alisa B 23-acetate (AB23A) (51)	+		Ldlr−/− mice	+		FXR, AB23A, CYP7A1, CYP8B1, SHP, CDCA, BSEP
			L02 cells		+	
Isoliquiritigenin (99)	+		HUVECs		+	SIRT6, TNF-α, NLRP3
Geniposide (100)	+		ApoE−/− mice	+		CXCL14
			RAW 264.7		+	
Guang Chen Pi (82)		+	RAW 264.7		+	SRA1, CD36, PPARγ, LXRα, SRB1, ABCG1, p38 MAPK, ERK1/2, JNK1/2, NF-κB p65, IKKα/β
Chinese agarwood (101)		+	ApoE−/− mice	+		ER stress, P-JNK, PPARγ, CD36
Salvia miltiorrhiza		+	ApoE−/− mice	+		COX-2, TNF-α, NF-κB
Tanshinone IIA (102)	+		HUVECs		+	
Quercetin (103)	+		ApoE−/− mice	+		FGF2
			VSMCs		+	
Andrographolide (104)	+		RAW 264.7		+	NF-κB, CEBPB, PPARG
Dihydrotanshinone I (105)	+		RAW 264.7		+	NF-α, IL-1β, and MCP-1
Baicalein (106)	+		HUVECs		+	AKT1, MAPK1, PIK3CA, JUN, TP53, SRC, EGFR, ESR1
Tanshinone IIA (107)	+		LDLR(−/−) mice	+		MFGE8, CX3CR1, MERTK, AXL, TYRO3
			RAW 264.7		+	
Ginkgetin (108)	+		HepG2		+	CDK2
Puerarin and Tanshinone IIA (109)	+		ApoE−/− mice	+		HIF-1α, IL-1β
			HUVECs		+	
			THP-1		+	
Carthamus tinctorius L. (110)		+	ApoE−/− mice	+		IL-1β, IL-6, CXCL12, miR166a-3p, ICAM-1, VCAM-1
			HUVECs		+	
Evodiamine (111)	+		LDLR−/− mice	+		PCNA, MMP2, Myh11, Acta2, Nrf2, TNF-α, IL-1β, PI3K, Akt
			MOVAS		+	
Diterpenoids (112)	+		RAW264.7		+	ABCA1/G1, LXRα, PPARγ
Tribulus terrestris L. (113)		+	ApoE−/− mice	+		α-SMA, OPN, Akt, MAPKs, MEK, ERK, JNK
			A7r5		+	
Naringenin and quercetin (114)	+		RAW264.7		+	VEGFA, MMP9, IL-1β
Triptolide (115)	+		HUVECs		+	IκBα, NF-kappa B
Cryptotanshinone, isoeugenol, tanshinone IIA (116)	+		SD rats	+		Akt, p-Akt, ERK1/2, p-ERK1/2, cSrc, p-cSrc, RhoA
Ganoderma lucidum spore powder (117)		+	LDLR(−/−) mice	+		ABCA1/G1, RUNX2
Salvianolic acid A (118)	+		ApoE−/− mice	+		PKR, PKM2
Rhodiola rosea polysaccharides (119)	+		Balb/c mice	+		PI3K, AKT, GSK-3β

**Figure 3:**
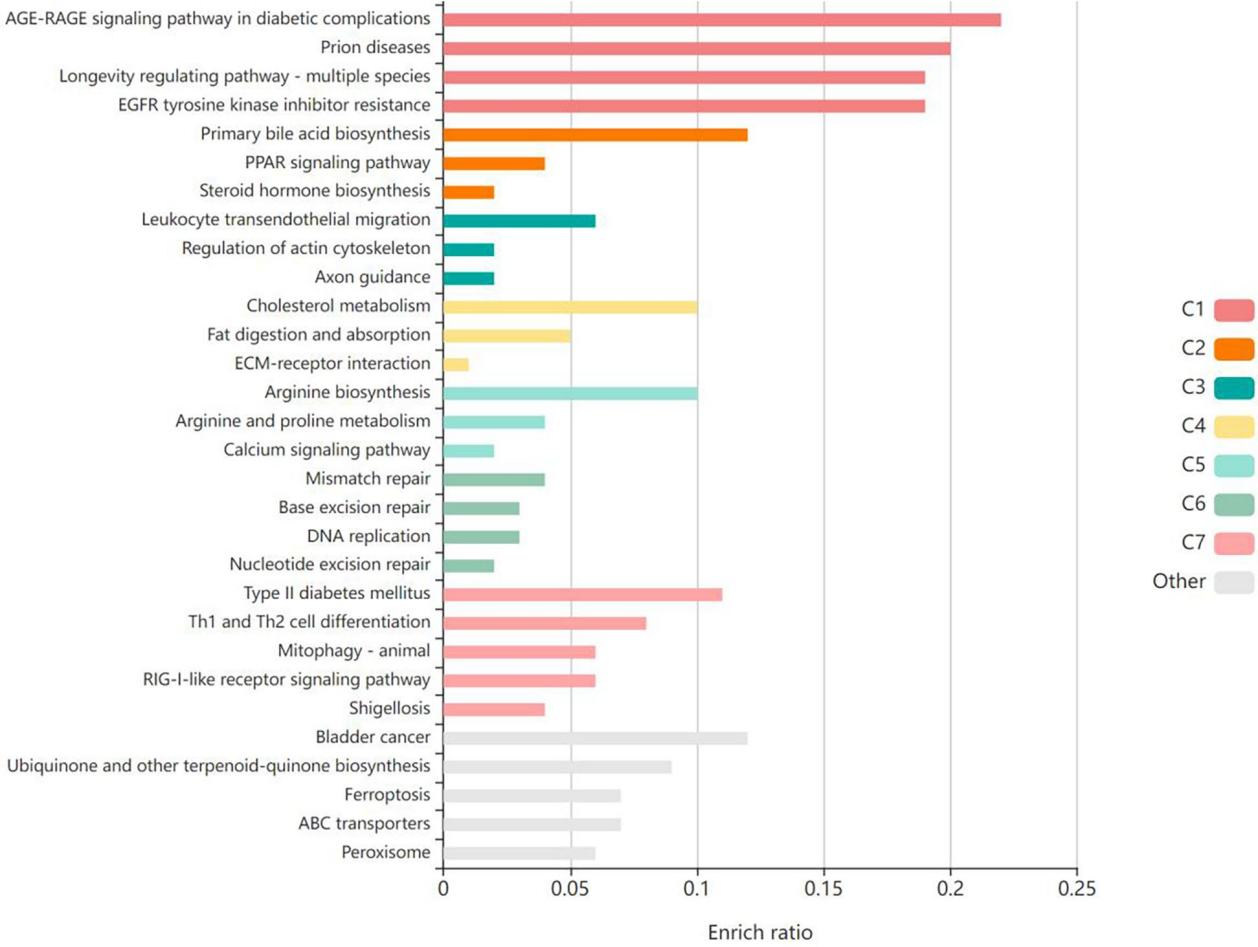
The validation targets were enriched in GO biological processes, and the top 30 terms were significantly enriched. Produced by KOBAS (http://kobas.cbi.pku.edu.cn/).

**Figure 4:**
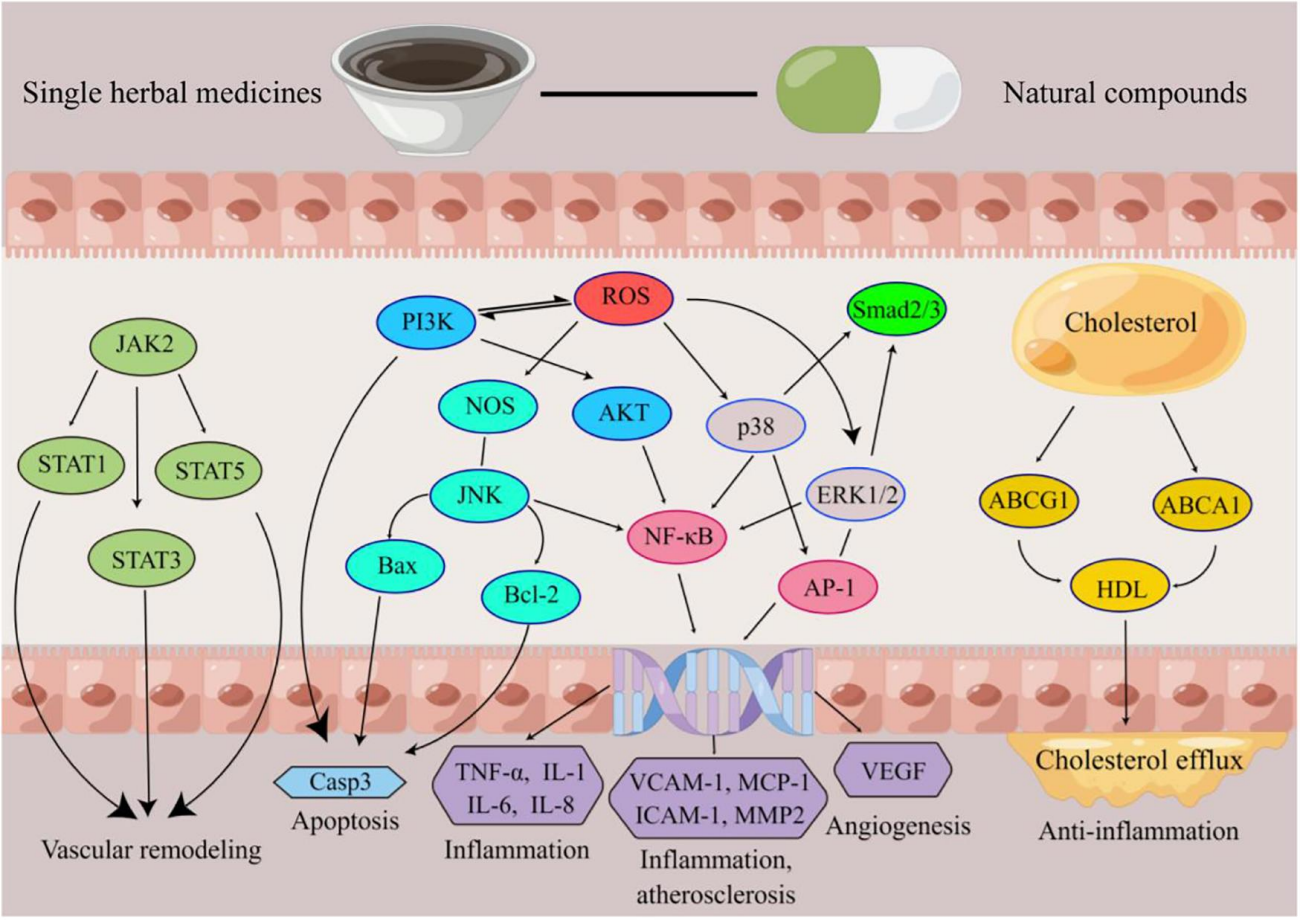
Schematic diagram of the mechanism of action of Chinese medicine for AS. Produced by Figdraw (https://www.figdraw.com).

The validation targets were enriched in GO biological processes, and a total of 207 terms were enriched, of which 30 terms were significantly enriched (Figure 3). Among them, the most obviously enriched pathways were the AGE-RAGE signaling pathway in diabetic complications, prion diseases, the longevity regulating pathway in multiple species, EGFR tyrosine kinase inhibitor resistance, and the adipocytokine signaling pathway. Through the description of these five terms by KEGG, we can learn that the main functions of these terms are related to inflammation, glucose metabolism, lipid metabolism, autophagy, and apoptosis, which often profoundly affect the generation and development of AS. The AGE-RAGE signaling pathway in diabetic complications activates several intracellular signaling pathways that can lead to NF-κB activity. NF-κB promotes the expression of a series of proinflammatory cytokines and various atherosclerosis-related genes, such as VCAM-1, VEGF, and RAGE. In addition, the AGE-RAGE signaling pathway is involved in cell proliferation and apoptosis in diabetic patients. Pathways of action in prion diseases include oxidative stress, the regulatory activation of complement, the corticosteroid response and endoplasmic reticulum stress. In longevity-regulating pathways, multiple species are involved in antioxidative stress, energy metabolism, DNA damage repair, glucose metabolism, autophagy, and other processes, thus resisting molecular, cellular, and organ damage; preventing the loss of various functions of human tissues; and reducing the risk of disease and death. EGFR can effectively interfere with the growth, differentiation, and proliferation of vascular cells. The adipocytokine signaling pathway is an important regulatory pathway for energy intake and metabolic rates. This function is due mainly to JAK kinase activity, STAT3 phosphorylation, and activation of AMPK to inhibit endogenous glucose production. In addition, JNK, mTOR, and IKK have also been shown to be involved (Figure 4).

#### 3.1.1 Inflammation

Inflammation is the most significant risk factor for AS. The development of vascular inflammation is mainly due to the expression of various effector proteins, including adhesion molecules [e.g., intercellular adhesion molecule-1 (ICAM-1) and VCAM-1 (VCAM-1)] and chemokines [e.g., IL-1, IL-8, and monocyte chemoattractant protein-1 (MCP-1)]. Abnormal expression of these proteins leads to vascular endothelial cell injury and dysfunction.

In the area of anti-inflammation, numerous studies have demonstrated the superior efficacy of Chinese medicine. The anti-inflammatory properties of cryptotanshinone may prevent and treat cytokine-induced atherosclerosis (5). The mechanism of action of lactones from Lilusticum chuanxiong Hort. lactones may be related to the inhibition of NF-κB expression (75); this mechanism is very similar to that of coptisine, but coptisine also acts through the MAPK signaling pathway (48). Many other medicines, such as leonurine from Leonuri (45), polygoni multiflori radix and TSG (93), and Danshen (83), have demonstrated antiatherosclerotic effects associated with inflammation.

#### 3.1.2 Lipids and glucose

Hyperlipidemia is also an important risk factor for AS and is characterized by elevated serum levels of total cholesterol (TC), triglycerides (TG), and LDL-C. For hyperlipidemia, statins are currently the mainstay of treatment. However, owing to the side effects of statins, such as liver damage, the search for new alternative drugs remains an important research direction. High glucose can interfere with the endothelial cell cycle, increase endothelial cell injury, delay endothelial cell repair, and cause excessive cell death. In addition, high glucose can lead to structural destruction of the vascular endothelium through oxidative stress, the production of large amounts of advanced glycation end products (AGEs), and damage to endothelial progenitor cells. Damage to endothelial cells and disruption of their structure impair their normal function, which in turn leads to AS.

Some natural lipid-lowering compounds, such as icariin (37), baicalin (40), Ganodermalucidum triterpenoids and polysaccharides (43), coptisine (48), astragaloside IV (65), and disocin (120), are also useful. Many Chinese medicines, such as resveratrol (26), ophiopogon polysaccharide, notoginseng total saponins, Rhizoma coptidis alkaloids (41), baicalin, baicalein, and wogonin (56), which have significant therapeutic effects on hyperglycemia, diabetic complications, and atherosclerosis, have excellent hypoglycemic effects. However, they do not have the same mechanism of action. The AMPK/ACC pathway, Akt/GSK-3β pathway, and VEGF/KDR pathway are involved. The AMPK/ACC pathway is a key influencer of adipogenesis, the Akt/GSK-3β pathway is a key influencer of glycogen synthesis, the VEGF/KDR pathway ameliorates caveolae-mediated hyperpermeability induced by high glucose, and the endothelial hyperpermeability induced by hyperglycemia is an important prerequisite for the development of AS. There are also medicines, such as Rhodiola rosea polysaccharides (121), that have excellent modulatory effects on blood glucose and blood lipids. Because the mechanism of action is not described in the article on R. rosea polysaccharides, we have not cited it in the table. However, other studies have shown that Rhodiola rosea polysaccharides can increase hepatic glycogen synthesis and improve hepatic glycogen metabolism in diabetic mice by regulating the PI3K/AKT/GSK3β pathway in the liver. Those study confirmed the hypoglycemic and hypolipidemic effects of this natural compound and its great industrial value.

#### 3.1.3 Endothelial cells (ECs)

The vascular endothelium is the continuous cellular lining of the cardiovascular system and is a key link in the development of numerous vascular problems, such as vascular tone, thrombosis, VSMC proliferation, leukocyte adhesion, and vascular inflammation. In recent years, endothelial function impairment has been found to be the primary and earliest link in the development of AS. The earliest manifestation of AS is impaired diastolic function when there are no microscopically visible lesions in the endothelium or vascular wall, and the only changes are decreased release of nitric oxide (NO), increased peroxides, and increased oxygen ions, which are only signs of functional damage. Additionally, as a mechanical barrier between the vessel wall and plasma molecules, the endothelium plays a crucial role in maintaining vascular status, and endothelial cell dysfunction (ECD) is a major contributing factor to the progression of atherosclerotic plaques in the early stages of AS (122). The recruitment of inflammatory cells, which in turn leads to an inflammatory response in the vasculature, is an important factor leading to endothelial cell damage and dysfunction. Fortunately, many Chinese medicines have therapeutic effects on vascular damage and related inflammatory conditions.

Many Chinese medicines can treat ECD. The therapeutic effect of ginsenoside Rb2 is achieved by targeting miR-216a (7). 13-Methylberberine has significant hypolipidemic and anti-inflammatory activities. In addition, this function was achieved by inducing autophagy in HUVECs and inhibiting the activation of NLRP3 inflammatory vesicles, thus exerting cytoprotective effects in a model of H2O2-induced injury in HUVECs (21). Triptolide may inhibit the inflammatory response of endothelial cells by inhibiting NF-κ B activation (23). The PI3K/Akt signaling pathway plays a key role in total saponins of aralia elat of promoting cell survival and anti-inflammatory responses (36). A key component of the therapeutic effect of ganoderma lucidum triterpenoids and polysaccharides is the regulation of the Notch1 and DLL4 pathways (43). The therapeutic effect of kaempferol is associated with activation of the GPER-mediated PI3K/AKT/Nrf2 pathway (50). Other medicines are dendrobium ling zhi 8 (42), ganoderma triterpenoids (54), artesunate (55), disocin (77), catenatum lindl (81), cynanchum wilfordii (90), all of which have similar functions, although the mechanism of action varies. The therapeutic effects of salvianolic acid B (15), scutellarin (44), tanshinone IIA sodium sulfonate (80), and extract of ophiopogonis radix (84) were reflected in the effects on ROS, NO, eNOS, and MDA expression levels. Among them, the investigators found that scutellarin acted in a more diverse manner, with scutellarin restoring mRNA and protein expression of SOD1 and Nox4 in cellular experiments.

#### 3.1.4 Macrophages

As a chronic inflammatory response recognized by the medical research community, macrophages play an important role in the development of AS. Macrophages can phagocytose inflammatory cells and actively participate in cholesterol accumulation, on the other hand, anti-inflammatory macrophages contribute to tissue repair and plaque stabilization.

Many Chinese medicines have been shown to inhibit foam cell formation and cholesterol accumulation in RAW264.7 macrophages, but the mechanisms by which they exert anti-AS effects vary. Clematichinenoside AR (8) acts through upregulation of ABCA1/ABCG1, gypenoside (123) acts by enhancing Sirt1-FOXO1-mediated autophagic flux, tanshindiol C (30) acts by activating the Prdx1/ABCA1 signaling pathway, celastrol (52) acts by inhibiting the production of LOX-1 and ROS. Additionally, Guang Chen Pi (82), Danshen (92) had the same effect. The functions of saikosaponins-a (12), curcumin (18) in promoting cholesterol efflux from macrophage-derived foam cells were similarly demonstrated on THP-1 macrophages.

Some Chinese medicines achieve their anti-AS effects by regulating the different polarization patterns of M1-type macrophages and M2-type macrophages. Leech peptide HE-D (27), catalpol (46), tanshinone IIA (47), artesunate (72) inhibit M1 subtype macrophage polarization, while providing enhanced M2 macrophage polarization. Some representative mechanisms of action are leech peptide HE-D (27) which can act by decreasing the expression of IKK α and IKK γ in the NF-κ B signaling pathway, catalpol (46) which acts by increasing the expression of ER α, tanshinone IIA (47) which acts by inhibiting miR-375 and activating KLF4, and artesunate (72) which acts by regulating HIF-1 α and NF- κB signaling pathways.

In addition to *in vitro* experiments, numerous *in vivo* experiments have provided evidence that Chinese medicines affect macrophages. Berberine (62) reduces macrophage activation as well as cytokine release, and celosins (69) inhibit lipid phagocytosis by macrophages while increasing intracellular autophagy levels through increases in LC3 and Beclin-1. Eucommia leaf extract (91) can modulate the function of macrophages and reduce the number of AS lesions.

#### 3.1.5 Vascular smooth muscle cells

VSMCs are located in the middle membrane of the vessel wall. In response to growth factors and proinflammatory factors secreted by foam cells, VSMCs proliferate and migrate to the middle membrane of the vessel and participate in plaque formation together with foam cells and macrophages.

Numerous studies have shown that different Chinese medicines can significantly inhibit the proliferation and migration of VSMCs and promote their apoptosis, although their mechanisms of action are different. Evodiamine (10) works by upregulating PPAR, afrocyclamine A (11) acts through the p38 MAPK signaling pathway, (2S)-naringenin from Typha angustata (16) acts by blocking G(0)/G(1), protocatechuic aldehyde (20) acts through antioxidants, celastrol (22) acts by activating LXR α and upregulating ABCA1, hydroxysafflor yellow A (25) acts through the akt signaling pathway, total flavonoids from dracocephalum moldavica (28) act by reducing the expression of VCAM-1, ICAM-1, NF-κ B p65, PCNA, and Lycium barbarum polysaccharide (32)s act through the PI3K/Akt signaling pathway, and shikonin (33) has a wide range of regulatory effects on the NF-kappa B, Bax, Bcl-2, cyclin D1, cyclin E, caspase-3, caspase-9, and PI3K signaling pathways, with hyperoside (34) acting through the LOX-1-ERK pathway.

#### 3.1.6 Platelets

In the treatment of AS, antiplatelet drugs are receiving increasing attention, and their proper use can prevent platelet aggregation and thrombosis. Recent studies have shown that platelets act as “inflammatory cells”, and their activation releases some inflammatory mediators that are directly involved in the formation and development of atherosclerosis and are closely related to plaque instability. We focus on the role of Chinese medicines in preventing platelet aggregation and thrombosis. Protocatechuic aldehyde (20) has been shown to inhibit platelet aggregation and prevent thrombosis in studies, whereas leonurine (45) acts by reducing the expression of platelet-endothelial cell adhesion molecule-1 (PECAM-1) in the aorta. Astragaloside IV (65) not only reduces platelet aggregation but also inhibits the expression of CXCR4, CD40l, and PAC-1 on the platelet surface. Baicalin (124) also has significant antiplatelet aggregation effects, but the exact mechanism has not been investigated in depth by the authors.

#### 3.1.7 Dendritic cells

In a high-fat environment, abnormal lipid metabolism in dendritic cells (DCs) leads to abnormal immune function, promotes the development of immune inflammatory responses, and facilitates the development of AS. Baicalin and geniposide (59) can reduce atherosclerotic lesions and modulate bone marrow dendritic cells, and alisol B 23-acetate (68) promotes cholesterol efflux from DCs and reduces the expression of CD80, CD86, and MHC II and the production of the inflammatory cytokines IFN-γ and IL-12.

### 3.2 Clinical studies

#### 3.2.1 Research overview

**Figure 5:**
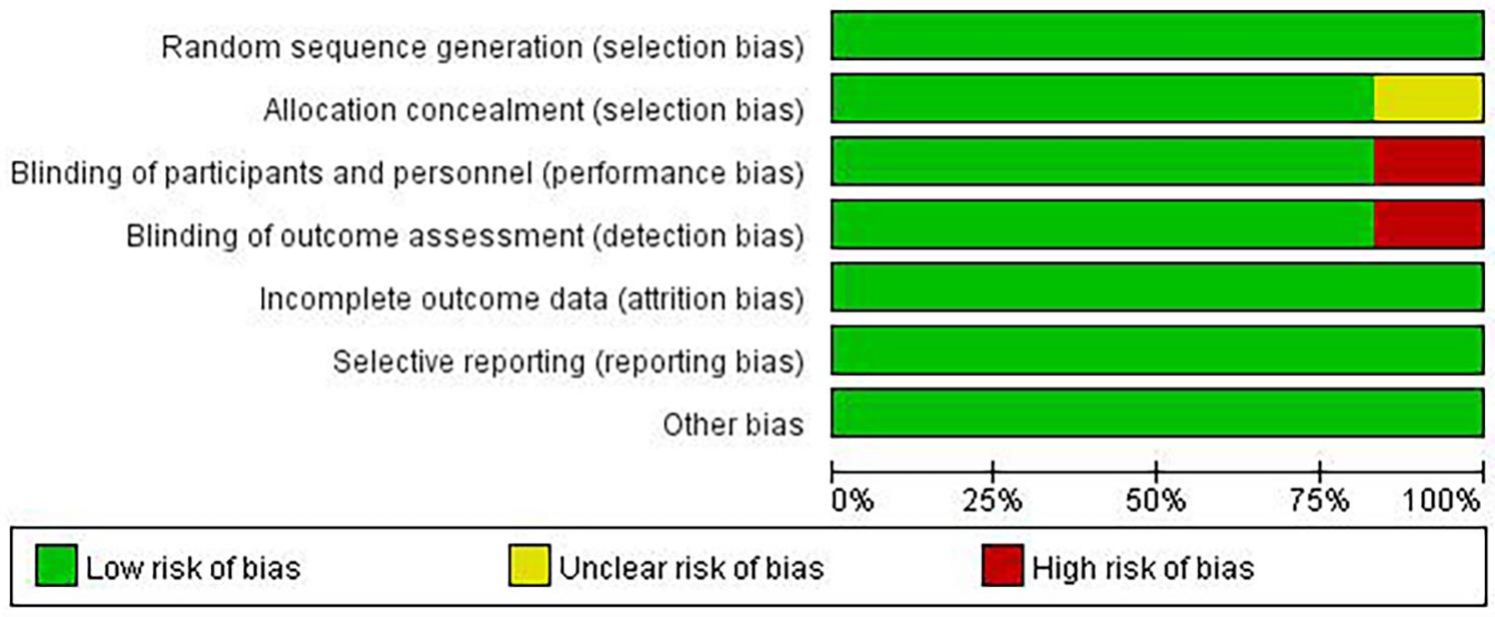
Risk of bias summary.

**Figure 6:**
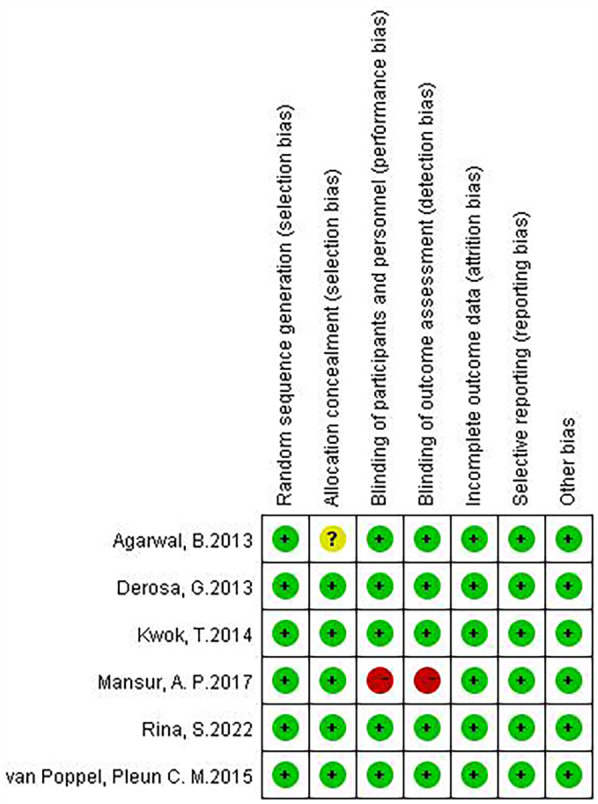
Risk of bias graph.

Although there are few clinical studies on Chinese medicines for the treatment of AS, we still found 6 relevant clinical research articles in addition to a large number of *in vivo* and *in vitro* studies in the course of our systematic review. These six articles focus on studies that address AS or AS-related high-risk factors, such as inflammation, lipids, and glucose. We then conducted an in-depth evaluation of these six articles. All studies were designed with at least one experimental group and one control group. Patients in the experimental group were treated with one or more single herbal medicines or natural compounds. Patients in the control group received only placebo or conventional treatment, which included exercise and lifestyle modification. All six included studies randomized patient groups, and the method of random assignment had a low impact on trial outcomes and was rated as “low risk”. Five studies had no risk of bias in allocation concealment and were rated as “low risk”, whereas one study did not explicitly mention allocation concealment and was rated as “unclear”. Five studies specified that double-blinding was used during the study to avoid implementation bias and detection bias and were rated as “low risk”, whereas one study did not mention blinding according to the article description and was rated as “high risk” for implementation bias and detection bias. Six studies were rated as “low risk” for the presence or absence of complete reporting of data-complete outcome indicators. All six studies were rated as “low risk” for selective reporting. No other risk of bias was found in any of the six studies, and they were rated as “low risk” (Figures 5, 6).

#### 3.2.2 Study results

The findings of these clinical studies reaffirm that Chinese medicine is stable and reliable for both AS and AS-related high-risk factors. A double-blind, randomized, placebo-controlled clinical study of RESV (400 mg of trans-resveratrol, 400 mg of grape skin extract and 100 mg of quercetin) revealed that RESV significantly reduced the mRNA expression levels of ICAM, VCAM and IL-8, as did the levels of inflammatory markers, such as plasma IFN-γ and insulin (125). A double-blind, randomized, placebo-controlled clinical study of berberine revealed that berberine reduced total cholesterol, triglyceride and LDL cholesterol and increased HDL cholesterol (126). A randomized, double-blind, placebo-controlled clinical study of Danshen (Salvia miltiorrhiza) and Gegen (Radix puerariae) (D&G) revealed that D&G significantly reduced LDL, cholesterol, and total cholesterol levels, as well as carotid intima-media thickness (IMT) (127). A multicenter, double-blind, double-model, positive-controlled, parallel-randomized controlled clinical study of *Ginkgo biloba* extract (GBE50) revealed that the TCM symptom pattern score was greater overall than the control group was. The TCM symptom pattern score was the main observation of this study (128). However, two studies have also shown that the effects of Chinese medicine are not consistent. A randomized, parallel, prospective clinical study revealed that resveratrol increased serum concentrations of Sirt1 but had no significant effect on metabolic pathways and that the drug increased total cholesterol and apolipoprotein B concentrations and HOMA-IR scores in subjects during the study (129). A randomized, placebo-controlled, double-blind crossover study of Salvia miltiorrhiza root water extract (Danshen) revealed that the drug mildly elevated LDL-C levels in subjects but had no effect on multiple other risk indicators for AS (130). The actual effect of Chinese medicine in clinical use is clearly not stable, and the current research on the causes of this instability is insufficient (Table 2).

**Table 2:** Clinical study of Chinese medicine for AS.

Name of Chinese Medicine	Type of Chinese Medicine		Number of patients	Patient Information	Results
	NC	HM			
Trans-resveratrol, grapeskin extract, quercetin (125)	+		44	Exclusion criteria: age <18 years; history of significant general illness; history of drug or supplement use that may alter metabolism or cardiovascular physiology; and use of grape-related supplements within 1 year.	Two subjects in the experimental group and one subject in the placebo group reported minor gastrointestinal side effects. For the experimental group, mRNA expression of ICAM, VCAM and IL-8 was significantly reduced (*p* < 0. 05), and biomarkers, such as plasma IFN-γ and insulin, also appeared to be reduced.
Derberine (126)	+		144	Caucasian subjects	Treatment with berberine for 3 months reduced total cholesterol, triglycerides and LDL cholesterol and increased HDL cholesterol compared to placebo.
Danshen (Salvia miltiorrhiza) and Gegen (Radix puerariae) (127)		+	165	165 women aged 47–65 years who had been menopausal for more than 12 months were included in the study.	There were no significant changes in blood pressure and general biochemical parameters in both groups and serum LDL, cholesterol and total cholesterol were significantly lower in the experimental group. Carotid intima-media thickness (IMT) decreased by 1.52% from baseline in the experimental group, while it decreased by only 1.13% in the placebo-treated group. Twelve adverse events were reported (6 in the placebo group and 6 in the experimental group), but none were directly related to the study's herbal formulation.
*Ginkgo biloba* extract (GBE50) (128)	+		404	404 patients with cerebral atherosclerotic dizziness (blood stasis symptom pattern) from 10 hospitals in China	The overall efficiency of the experimental group was higher than that of the control group after 6 weeks of treatment (TCM symptom pattern score). The difference in the incidence of adverse reactions between the experimental group and the control group was not statistically significant. One drug-related adverse event occurred in the experimental group, showing mild elevation of ALT, AST and GGT, which was relieved after discontinuation of concomitant drugs (statins). One serious adverse event occurred in the control group.
Resveratrol (129)	+		48	Twenty-four women with 1-year spontaneous amenorrhea and 24 men between the ages of 55 and 65 years.	Resveratrol increased the concentration of Sirt1, with insignificant effects on various metabolic pathways. In contrast, total cholesterol and apolipoprotein B concentrations and HOMA-IR scores increased after treatment.
Danshen (130)		+	20	Subjects with hyperlipidemia and hypertension between the ages of 40 and 70 years. Patients on antihypertensive medication were included as eligible when the average of three office blood pressure recordings (sphygmomanometer, sitting position, at least 5 min apart) showed a systolic blood pressure >140 mmHg and/or a diastolic blood pressure >90 mmHg. Subjects with fasting LDL cholesterol >3.5 mmol/L and/or triglycerides >1.7 mmol/L. Excluded subjects were those with triglycerides >8 mmol/L and/or LDL-cholesterol >5 mmol/L or systolic blood pressure >180 mmHg and/or diastolic blood pressure >110 mmHg.	Mild increase in LDL-cholesterol after 4 weeks of Danshen (water-extract) treatment, no change in various other risk indicators.

The incidence of adverse events in these clinical studies was 0%–10% (125–130). Two RESV (400 mg trans-resveratrol, 400 mg grapeskin extract, and 100 mg quercetin) subjects reported mild gastrointestinal side effects (125). Regarding the study of “GBE50”, one drug-related adverse event occurred in the experimental group, but had no relation with traditional Chinese medicine (128), No adverse reactions were reported in other clinical studies.

## 4 Discussion

**Figure 7:**
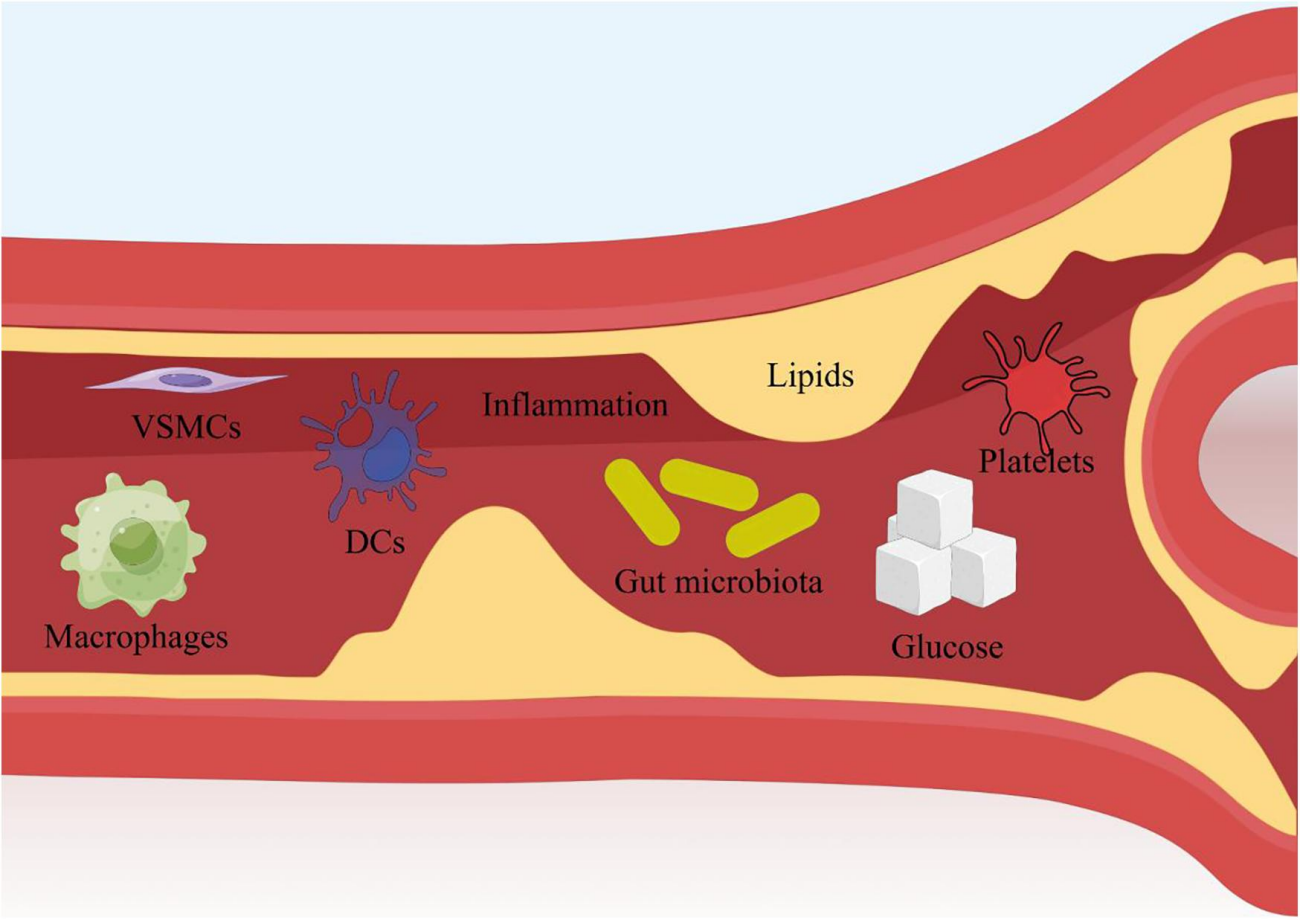
The main methods of Chinese medicine for treating AS. Produced by Figdraw (https://www.figdraw.com).

This review systematically integrates preclinical and clinical evidence to demonstrate that single herbal medicines (SHMs) and their derived natural compounds (NCs) exert anti-atherosclerotic (AS) effects through multifaceted mechanisms targeting key pathological drivers of AS. As summarized in Figure 7, Chinese medicines act on critical cellular components (e.g., endothelial cells, macrophages, vascular smooth muscle cells) and molecular pathways (e.g., NF-κB,MAPK, PI3K/Akt), underscoring their multi-target, multi-pathway regulatory characteristics. Preclinical studies highlight that TCM targets a spectrum of molecular nodes, Clinical trials further validate these effects. Collectively, these findings establish TCM as a promising therapeutic strategy for AS by concurrently targeting multiple pathological pathways.

Despite promising preclinical and clinical data, three critical challenges hinder TCM's translation into robust AS therapies. The first is efficacy instability. Clinical studies report inconsistent outcomes. For example, resveratrol failed to improve metabolic pathways and elevated total cholesterol in some trials. Such variability may arise from heterogeneous study designs (e.g., dosage, duration), population differences, or unstandardized TCM preparations. The lack of deep mechanistic exploration for these discrepancies limits our ability to optimize clinical application. The second is the mechanistic blind spots. While preclinical studies have identified numerous targets and pathways, a systematic understanding of how TCM components coordinate to exert holistic effects remains elusive. Most research focuses on single-target or single-pathway analyses, neglecting network-level interactions. Additionally, the dynamic temporal and spatial regulation of TCM *in vivo*—such as bioavailability, metabolite profiling, and tissue-specific effects—requires further clarification. Finally, Chinese medicine has good safety at specific doses, but potential risks need to be paid attention toIf. Although clinical trials report low adverse event rates (0%–10%), potential risks are underexplored. Basic research rarely investigates long-term toxicity, drug-drug interactions, or dose-dependent side effects. This gap raises concerns about long-term clinical use.

In order to address these challenges, improve the efficacy stability and safety of TCM in treating AS, and clarify the blind spots at the mechanism level, we propose that future research can focus on the following directions. To enhance therapeutic consistency, we can implement modern techniques such as high-performance liquid chromatography (HPLC) fingerprint analysis and biomarker quantification to standardize quality control of traditional Chinese medicinal materials, thereby minimizing fluctuations in active ingredient concentrations. By designing multi-center randomized double-blind controlled trials (RCTs) stratified by patient groups (classified by AS severity and comorbidity types), we can precisely identify subgroups with optimal therapeutic outcomes for TCM treatments. Concurrently, pharmacokinetic-pharmacodynamic (PK-PD) models are employed to optimize dosing regimens, enabling precise therapeutic regulation. To clarify the blind spots of mechanisms, through integrate multi-omics (genomics, proteomics, metabolomics) to map TCM's global regulatory networks, identifying key hubs and crosstalk between pathways. Adopt advanced Models, utilize organ-on-a-chip or humanized mouse models to simulate AS progression *in vivo*, enabling real-time tracking of TCM's spatiotemporal effects. To strengthen the safety evaluation, we can conduct long-term (6–12 months) toxicity studies in relevant animal models, focusing on liver, kidney, and gastrointestinal systems. Establish standardized adverse event reporting protocols in TCM-AS trials, with specific attention to rare but severe reactions (e.g., hepatotoxicity). Drug interaction assessment is also required, evaluate potential interactions between TCM and conventional AS drugs (e.g., statins) to prevent adverse synergies or antagonisms.

In summary, while traditional Chinese medicine has historically addressed AS-related symptoms without explicit terminology, modern research confirms its scientific basis for targeting AS pathogenesis. However, translating this potential into clinical practice requires rigorous, evidence-driven efforts to resolve efficacy instability, decode mechanisms, and ensure safety-steps that will ultimately validate and optimize Chinese medicine as a key player in AS prevention and treatment. In conclusion, although the modern name of AS has never been explicitly proposed in traditional Chinese medicine owing to the limitations of diagnostic techniques and equipment, it has consciously or unconsciously provided ideas and inspiration for how to treat AS in the modern context in the clinical practice of traditional Chinese medicine for thousands of years. Many single herbal medicines and natural compounds have indeed proven effective in treating AS. However, much work is still needed to improve how to make efficient use of these valuable experiences.

## Data Availability

The original contributions presented in the study are included in the article/Supplementary Material, further inquiries can be directed to the corresponding authors.
